# Refractory chronic cough, or the need to focus on the relationship between the larynx and the esophagus

**DOI:** 10.1186/1745-9974-9-10

**Published:** 2013-04-03

**Authors:** Adalberto Pacheco, Ignacio Cobeta

**Affiliations:** 1 Chronic Cough Unit, Pneumology Service, Hospital Ramón y Cajal, Madrid, Spain; 2 Otolaryngology Department, Hospital Ramón y Cajal, Madrid, Spain

## Abstract

In this review we question the current way of handling tackle a problem of chronic cough, especially by the excessive number of patients who can not find complete relief from your cough by anatomical diagnosis of universal use. From the field of Otolaryngology new perspectives arise now considering the larynx as a preferential afferent stimuli cough reflex arc. Also the constitution laryngopharyngeal reflux gas and new approaches to non-acid reflux and the local action of pepsin in laryngeal deserving of a joint review, which can illuminate new ways to handle the problem of chronic refractory cough. We believe that the chronic cough syndrome hpersensitivity as more precise label for chronic cough, should place particular emphasis on laryngeal sensory neuropathy as cough and reflux the influence that may have on their maintenance, and thereby causes definitely wide related to the syndrome if the larynx is incorporated, place greater number of afferent nerves of chronic cough, which are sure to cover much of the case of refractory cough remain without a satisfactory solution. The close collaboration between Otolaryngology, Gastroenterology and Pneumology in a patient with refractory chronic cough seems now an unavoidable necessity.

## Introduction

Cough is the final result of a vagally-mediated reflex and is part of a vegetative vagal function including swallowing and voice. Cough has a homeostatic function but it may also become a source of problems. Chronic cough (CC) affects as many as 10% of the general population [1]. The clinical diagnosis of chronic cough is based on cause and effect in conditions such as asthma, gastroesophageal reflux (GER) and upper airway syndrome, but not in diseases such as COPD, cancer, and heart failure which do not necessarily imply cause and effect [2]. In cases of cause-effect CC, after ruling out eosinophilic airway inflammation (asthmatic or non-asthmatic eosinophilic bronchitis) there is growing evidence that the most important etiology is a sensory disorder of the laryngeal branches of the vagus nerve. In these cases laryngopharyngeal reflux (LPR) is often concurrently diagnosed [3,4]. This is no surprise, since the vagus nerve supplies the entire aerodigestive tract, including the upper and lower respiratory tracts and the digestive tract. The present review focuses on the relationship between sensory vagal neuropathy and LPR and its impact on the treatment of refractory CC.

Upper airway cough syndrome (UACS), an important component of the diagnostic triad in CC, is of interest to three medical specialties: otolaryngology (ENT), pneumology and gastroenterology. Recently it has been the focus of attention in research into the diagnosis and treatment of refractory CC.

Unlike pulmonary CC patients, ENT patients with CC almost always have associated symptoms such as globus, dysphagia, dysphonia, dyspnea and/or stridor. However, although the onset of CC is associated with eosinophilic airway inflammation, it may also be associated with GER [5]. A problem still being debated in the CC literature is the fact that the condition may be the only (or the predominant) symptom of distinct pathologies located in different sites – for example, the upper airways, the lungs, or the digestive area – each one of which may trigger the cough reflex. Therefore, the temptation to describe a syndrome with a common denominator such as CC is very strong. In fact, when a common low threshold in the cough reflex was shown in relation to CC from many locations, all innervated by the vagus nerve, this finding led to the definition of Chronic Cough Hypersensitivity Syndrome (CCHS). Thus, CC is no longer a symptom, but has become a syndrome.

However, the prevalence of unexplained CC varies between clinics and in some studies has been reported to be as high as 42% [6]. This has led to calls for a new approach to chronic cough. For example, serious problems remain regarding the conceptualization of UACS, since the cohort of symptoms deriving from laryngeal neuropathy and /or LPR can be confused with those of extraesophageal reflux [7]. As a result, specific symptoms deriving from laryngeal conditions such as laryngospasm or paradoxical vocal fold motion (PVFM) are rarely considered in the CC guides [8,9]. The theory that CC predominantly initiates in the laryngeal area is gaining ground today, especially now that the triad of eosinophilic airway inflammation, GER and rhinitis-sinusitis has been ruled out. There is growing evidence that the larynx, the bridge area between esophagus and tracheo-bronchial tree, is one of the key sites of the afferent limb of the cough reflex, and may be especially important in cases of refractory CC. Recent reports of improvements in refractory cases of CC treated with speech pathology intervention are of great interest [10,11]. Diagnosis and treatment protocols for CC are usually produced by pneumologists [8,9]; however, ENT specialists have recently announced new perspectives in management and treatment [3], supporting the idea that a laryngeal neuropathy is responsible for many of the previously refractory cases of chronic cough. As the focus in studies of CC turns towards the larynx [3,12,13], pulmonologists are gradually losing their predominant position; or, more exactly, it is becoming more evident that CC requires close cooperation between these two specialties, and that gastroenterologists should also be involved in the development of protocols for treating the condition.

### Developments in the cough reflex arc

The afferent limb of the vagus nerve innervates diffuse structures of the aerodigestive tract and is responsible for the development of the cough reflex. These structures include the ear, pharyngeal branches, superior laryngeal branches, pulmonary branches, gastric branches from the stomach, cardial and esophageal branches and vagally innervated viscera. One of the methodological problems in basic research into CC is that the cough pathways in inflamed airways may be quite different from the ones observed in healthy airways. Airway sensory afferent nerves terminate in the brainstem at the level of the nucleus tractus solitarius [14]. In animal models it has been demonstrated that although general anesthesia may depress the respiratory center, the cough reflex remains intact.

There are at least five types of intraepithelial sensory receptors in the cough reflex. The most important are the rapidly adapting stretch receptors (RARs) and the bronchial C fiber receptor. In general, the distribution of the receptors is more mechanosensitive toward the larynx and more chemosensitive in the distal airway [12]. Current data support the hypothesis that at least two types of vagal nerves are responsible for initiating the cough reflex arc. One type is characterized by rapidly adapting responses to mechanical probing or acidification of the large airway epithelium. These nerves, which are capsaicin-insensitive, are important in immediate cough evoked by aspiration, and perform a critical role in airway defense. The other primary afferent nerve involved in cough is the capsaicin-sensitive vagal C-fiber. In clinical studies, inhalation of a low concentration of a C-fiber stimulant causes an irritating, itchy urge-to-cough sensation associated with respiratory tract infection, gastroesophageal reflux disorders, and inflammatory airway diseases [15]. Interestingly, acidification is a trigger in both types of afferent nerve.

Also important is the finding that some C-fibers may be linked to an inhibition of the cough reflex evoked by inflammatory mediators. Two strategies are currently proposed for the treatment of cough: decreasing the generator potential amplitude by acting on TRPV1 and TRPA1, and decreasing the efficiency with which a generator potential evokes the action-potential discharge (the voltage-gated sodium (NaV) channel), which is activated when the depolarization is of sufficient magnitude for the channel to open and the action potential discharge to be initiated [16].

However, the overlapping responsiveness of the capsaicin-sensitive and -insensitive vagal afferent nerves regulating cough argues against the validity of this distinction [15,17]. Cough should be seen as a phenomenon with a multifactorial origin; it should be accepted that the presence of a single origin is rare, and in fact the idea of different simultaneous locations is gaining support. According to Canning et al., it is apparent that multiple afferent nerve subtypes act in concert to regulate the sensitivity, forcefulness, and repetition of cough in response to all tussive stimuli. These authors hypothesize that alterations in cough reflex sensitivity may be attributable to alterations in the excitability of both afferent nerve subtypes and that, as a result, therapies targeting either pathway may provide benefit [18]. It is now accepted that a single stimulus – for example bronchospasm, acid in the esophagus or upper airway challenges with a variety of inflammatory mediators – is consistently ineffective in initiating cough in animals or human subjects and that the integration of the stimuli of several afferent limbs of the vagus nerve is necessary. There is considerable evidence of convergent vagal afferent nerve subpopulations relaying neurons in the nucleus of the solitary tract. This convergence may account for the imprecision of the visceral reflexes; the activation of a reflective body initiates changes in output flow to other autonomous organs, as Mazzone et al. demonstrated in guinea pigs [19]. This may also explain why the initial signal of the cough reflex is weak, but may be severe and chronic if reflex arc activation is recorded in several sites, as for example in the conjunction of eosinophilic airway inflammation and GER [5].

A good example of this concept is the role of reflux, as a precipitant of the cough reflex arc at several levels: not only the esophagus but upper respiratory and lower respiratory tracts [20]. However, there are two problems with this theory of a leading role for reflux: i) the question of whether GER causes cough, or vice versa, and ii) the lack of consensus on definitions in the extraesophageal area which may be affected by reflux. The functional diagnosis of GERD is verified in three ways: sustained distal acid esophageal exposure (pHmetry), microaspiration due to ineffective superior laryngeal sphincter (double pHmetry), or ineffective esophageal motility and prolonged acid clearance [21]. However, the nature of the reflux is important to diagnosis. In the case of CC from the upper airway, studies such as Kavamura et al. can be revealing; those authors showed that the gas with weak acidity reflux is more common among patients with laryngeal lesions than in patients with GERD or in controls [22]. Currently it seems of paramount importance to reach a consensus on the nature of the reflux (acid or non-acid, gas or liquid) and on the use of an upper airway pH of 5 as pathological threshold. Therefore new techniques like the Dx-pH measurement system (Dx-pH; Restech Corporation, San Diego, CA) which can measure the pH of pharyngeal gas reflux, and multichannel intraluminal impedance and pH testing (MII-pH) which can detect non-acid liquid reflux, should be implemented in the analysis of extraesophageal reflux [23].

One of the least explored associations of potential reflux provoking CC, apart from the consideration of non-acid reflux, is that of cough and esophageal dysmotility. This combination does not always respond to antacid therapy and is rarely analyzed in depth [24,25]. Moreover, it may represent a generalized abnormality of the neural aerodigestive network and, under a unified hypothesis, afferent vagal branches from the bronchus or upper airway may collaborate in the maintenance of CC. High-resolution manometry is recommended by the American Gastroenterology Association Institute to evaluate patients with suspected GERD syndrome who have not responded to empirical twice-daily PPI therapy and have normal endoscopic findings [26].

### CC as a laryngeal symptom

After ruling out the use of ACE inhibitors, smoking, COPD or altered chest X-ray, heightened cough reflex sensitivity is associated with eosinophilic airway inflammation, GER and UACS, the latter usually associated with rhinitis sinusitis in the CC guidelines [8,9]. In the larynx, cough reflex and laryngeal closure reflex coexist and play an important role both in protecting the airway during swallowing and also in response to noxious inhaled stimuli. The sensory nerve ending receptors which stimulate vocal cord closure and cough as part of the glottic closure reflex are found not only in the larynx but in the trachea and larger airways as well, and these may respond to pressure or to irritant stimuli. Hence, breathlessness is a relatively common finding in the clinical history of patients with laryngeal predominant symptoms plus CC [27]. However, triggers that stimulate the afferent limb of the cough reflex are now recognized to be an important part of the neurophysiology of cough, and in recent years it has been demonstrated that CC patients frequently report symptoms that suggest sensitization of the cough reflex and a neuropathic response. For example, after an acute episode of allergy, viruses, non-specific irritants or laryngopharyngeal reflux (LPR) a laryngeal sensory defect may develop and CC may remain as the most relevant symptom, though rarely the only one [3,11].

The three terms most frequently used in the literature and in clinical practice to refer to CC of predominantly laryngeal origin are paradoxical vocal fold movement (PVFM), laryngospasm, and vocal cord dysfunction (VCD). All these entities have a common basis, the permanent or intermittent closure of the glottic area. Many recent studies identify the at-risk population, the common presenting symptoms and potential pathophysiology underlying vagal neuropathy, and have proposed several promising medical interventions [11,28]. The relationship between CC and the laryngeal area has also been associated with states of hypersensitivity and hyposensitivity of the laryngeal mucosa. First introduced by Morrison and colleagues [29] in 1999, the concept of irritable larynx has been refined over the years, with various terms being used for the same suspected cause: postviral vagal neuropathy (PVVN), sensory neuropathic cough, and laryngeal sensory neuropathy. The considerable confusion in the terminology has probably added to the difficulty of treating these pathologies. Vagal neuropathy may affect the motor branches of the vagus nerve, resulting in vocal fold paralysis or paresis, or it may affect the sensory branches, inducing CC but also a throat tickle, globus sensation, excessive throat mucus, odynophonia, or laryngospasm. These symptoms may be aggravated by sensory stimuli such as laughing and prolonged phonation or noxious stimuli, and can be elicited clinically by palpation at the cricoid level. Some patients have psychogenic cough with an identifiable secondary gain or abrupt onset/offset of the problem.

A problem that may underlie the confusing diagnosis of refractory CC is the relative neglect of the symptomatic history prior to onset. Patients usually deny any associated symptoms or cough before the beginning of CC. Several aspects should be considered in the early stages. First, there may be several simultaneous sites of stimulation of the cough reflex. In one study [8] 62% of patients had two or more potential sites of origin, and in a study by our group of 270 patients with CC, 84% presented simultaneous sites [30]. Second, laryngeal paresthesias, that is, abnormal sensations, may be present in up to 94% of refractory CC [11]. Paresthesias and triggers of response to low level stimuli are features of neuropathic sensory defects or vagal neuralgia with central sensitization of the cough reflex. They have also received limited attention in the clinical research literature [31], so there is currently a need for objective measures of triggers and methods to identify, describe, and quantify laryngeal paresthesias [11].

### Laryngeal neuropathy and laryngopharyngeal reflux

A prominent characteristic of neuralgia is the presence of trigger phenomena. We speculate that a vagal laryngeal neuralgia may manifest as a sudden, exaggerated, but non-painful sensation in the vagus nerve that leads to uncontrollable coughing. The idea is that the threshold for the initiation of cough reflex is significantly lowered: thus, a strong, irresistible signal to cough could result from minimal stimuli, or from no stimulus at all. In 2001, Amin and Koufman [32] first described the association of CC and previous upper respiratory tract infection. Subsequent investigation through videostroboscopy and laryngeal electromyography revealed a variety of presentations: (1) vocal fold paresis, (2) neuropathy-induced laryngopharyngeal reflux disease, (3) dysphagia, and (4) neuropathic pain. In addition, the association of autosomal dominant hereditary sensory neuropathy with chronic cough and gastroesophageal reflux suggests a link between CC and sensory neuropathy mediated by gastroesophageal reflux [33,34].

The reason why only a few patients with upper respiratory infection develop chronic impairment of the laryngeal afferent branch remains a mystery. Two possible explanations have been proposed: the patient may develop chronic PVFM (laryngeal neuropathy); alternatively, he/she may simultaneously develop LPR, which can act in turn as chronic irritant on the larynx. Transient postviral vocal cord adduction has been demonstrated by Gibson et al. [35]. These authors argue that viruses infect the upper airway epithelium causing neural activation that can lead to either completely reversible or recurrent cough. PVFM may also be a motor response to laryngeal sensory neuropathy. It has been suggested that individuals with PVFM may cough willingly to cause vocal cord abduction during a PVFM episode [36]. However, given the frequent association of LPR and chronic PVFM, the hypothesis is that PVFM persists in the presence of LPR, with recurrent irritation of the larynx and pharynx.

GER may be clinically silent in as many 75% of patients referred for CC. Gastric refluxate has been hypothesized as a likely cause of the impaired laryngopharyngeal sensation in patients with CC and GERD compared to controls [3,13]. Decreased mechanosensitivity and chemosensitivity of the laryngeal mucosa due to chronic acid irritation could result in increased collection of particulate or irritant matter in the laryngeal area; the chronic cough reflex may simply be an adaptative mechanism to clear the larynx [3]. In an elegant paper, Murry et al. quantified the laryngeal sensory response in a cohort of patients with PVFM and CC who had received twice-daily proton pump inhibitors for at least three months with no subjective improvement, and demonstrated that mean bilateral laryngeal sensory response improved significantly after combined respiratory retraining and aggressive PPI therapy. All patients reported improved cough [3].

The most convincing explanation of the relationship between the states of hypersensitivity and hyposensitivity in the laryngeal mucosa was recently provided by Cukier-Blaj et al. [37]. These authors propose that laryngeal irritation caused by reflux decreases laryngeal sensitivity and results in a compensatory motor response with a hyperadduction of the vocal folds during inspiration, cough and dysphonia. This adaptive mechanism may explain the link between LPR and laryngeal neuropathy because if sensory deficits are prolonged in time, aberrant sensory repair regeneration or central changes may develop. This proposal may be supported by the recently reported success of respiratory retraining in patients with CC without complete resolution of cough [10,11].

This research in the field of ENT suggests that the chronic irritation of the larynx due to reflux may cause two conditions: first, a hyposensitive state with the risk of developing aspirations and cough; second, a hypersensitive state in which the larynx is more sensitive to various external stimuli [29]. Many patients report developing “sensitivity” to various non-specific triggers such as cigarette smoking, cold air, exercise, perfume, clearing agents, odors and emotional stress. This clinical presentation often leads to misdiagnosis of asthma or multiple chemical sensitivity. The disorder may be long-lasting as patients may be unsuccessfully treated for asthma and other comorbidities before correct diagnosis is made. Some patients present asthma but CC is usually associated with other LPR symptoms, episodic choking or shortness of breath, and often the breathing problems begin at the same time. In these cases LPR seems to be the cause of, or at least a decisive factor in, the breathing problems**.**

The endoscopic examination of the larynx is essential in all patients with CC since it is important to differentiate endoscopic signs of LPR from signs of vocal cord paresis [38]. It has recently been demonstrated that proximal non-acid reflux disease is more important in CC [39]. Using immunohistochemical analysis, Johnston et al. showed that 95% of LPR patients had tissue-bound pepsin within the tissue biopsy specimens, compared with only 5% of controls [40]. The same authors demonstrated that human pepsin retains some of its proteolytic activity up to a pH of 6.5 [41]. The speculation that refractory CC may be due to a form of non-acid reflux that is mainly gaseous, and is thus undetectable by conventional techniques such as esophageal pH monitoring or impedance, deserves more attention. In cases of CC associated with non-acid reflux, given the ineffectiveness of antacid treatment it may be advisable to use baclofen, a gamma-aminobutyric acid (GABA) B agonist which acts by decreasing the number of lower esophageal sphincter relaxations [42]. Moreover the recent discovery of proton pump activity in seromucinous glands of the human larynx opens up new possibilities not only for treatment but for identifying the pathogenesis of lesions in tissue laryngeal nerve endings, even without LPR [43].

These findings are clinically relevant in CC of laryngeal origin. First, the tissue-bound pepsin in these patients may be activated by any source of exogenous acid, including foods and beverages, and the activation of cough receptors in the larynx may perpetuate in spite of high doses of PPI and lifestyle modifications. Second, Altman’s study of the proton pump in the larynx did not contain enough cases to be able to establish the variability between individuals; it might be speculated that some patients with CC and with tissue-bound pepsin in the larynx could activate pepsin without the need for an external source. What is more, the proteolytic activity of pepsin in the larynx may result in sensitivity disorders such as glottic closure reflex and cough reflex.

### Chronic cough hypersensitivity syndrome

Chronic cough hypersensitivity syndrome (CCHS) is a syndrome that identifies a group of patients with unexplained chronic cough (UCC) after anatomical diagnosis of CC. Patients with UCC present abnormal cough reflex. These patients commonly have a strikingly increased sensitivity to cough challenge with capsaicin, which suggests an abnormality of the airway sensory nerves. Birring proposes a different term, “cough reflex hypersensitivity”, but we do not yet have a full exploratory method of the cough reflex arc covering the two main vagal afferent pathways, C fibers and cough receptors [44]. The sensory receptors that mediate cough in humans have not been conclusively identified, but members of the transient receptor potential (TRP) ion channel family are strong candidates. There is evidence of increased expression of TRPV-1 in the airway nerves of patients with unexplained chronic cough [45], and a recent study showed that airway inflammation can initiate the expression of TRPV1 and TRPA1 in airway afferent neurons that normally do not express these ion channels [46]. Future research should focus on important mechanisms such as cough reflex hypersensitivity. The symptoms usually associated with CCHS included persistent tickling or irritating sensation in the chest or throat, hoarse voice, dysphonia or vocal cord dysfunction (VCD) [2]. Heightened cough reflex sensitivity can be considered reversible or persistent in patients with CCHS in whom the cough is typically dry or minimally productive. Nevertheless, the hypersensitivity of the airway nerves may not be limited to those involved in cough, and so enhanced sensitivity of the laryngeal glottic closure reflex and the high prevalence of VCD and voice disorders in UCC raises the possibility of a generalized disorder of the airway nerves [10,47]. Abnormal sensation and triggering of responses with low-level or non-tussive stimuli are features of a neuropathic sensory disorder. Tussive triggers are those known to stimulate cough receptors, such as smoke and fumes, whereas non-tussive triggers are those not known to be tussigenic in neurophysiological testing, such as perfumes, cold air, vocalization, or stress. In addition, if an abnormal sensation in the laryngeal area occurs in the absence of exposure to a trigger, then the term used is laryngeal paresthesia (tickle, itching, mucous, tightness etc.). The triggers are recognized by approximately two thirds of patients referred with CC [31]; however, laryngeal paresthesia, as a part of the symptom profile of CC, was present in 94% of patients with CC which was refractory to medical management based on the anatomical diagnostic protocol [11].

Morice et al. maintain that the CC is part of a broader syndrome, termed CCHS. According to these authors, its pathophysiological basis is acid or non-acid liquid and gaseous reflux, and which comprises several phenotypes such as airway eosinophilic inflammation, GERD or upper airway involvement. The symptoms reported in the larynx are very common in this syndrome, and the authors justify their "unifying" hypothesis because of the greater density of laryngeal cough receptors. However, applying the Hull Cough Hypersensitivity Questionnaire, they also found evidence of a common history of all phenotypes of chronic cough [20]. McGarvey challenges this concept on the grounds that cough reflex hypersensitivity may not be present in all patients with CC and, in addition, successful treatment is usually (though not always) associated with reduced cough sensitivity [48]. However, one of the two types of the afferent nerve conducts of the cough reflex is characterized by rapidly adapting responses to acidification of the large airway epithelium; they are capsaicin-insensitive but may cause coughing, although no heightened response to tussive agents such as capsaicin has been detected [15]. In any case, we believe that if sensory alterations originating in the laryngeal mucosa are included in the diagnosis of CCHS, this establishes the concept of CC as an altered vagal reflex of multifactorial origin.

### Treatment of chronic cough of laryngeal origin

A long-term follow-up study by pneumologists of patients with UCC found that cough persisted in most patients but that its severity was reduced in more than 50% [49]. Another study by Vertigan AE et al. showed that CC which persists despite medical treatment may respond to speech pathology intervention; improvements were recorded in 88% of participants in the treatment group compared with 14% in the placebo group [10]. The importance of targeting sensation in CC to reduce the laryngeal adduction reflex in PVFM lies in the fact that it can improve with pharmacology and behavioral treatment. Voice therapies that prevent vocal fold hyperadduction are an important component of behavioral interventions in CC [10]. Respiratory retraining not only alters the respiratory mechanism, but also has the capacity to change the inherent laryngeal sensory response, probably raising the threshold of the cough reflex.

Most patients with CC have rhinosinusitis, eosinophilic airway inflammation, allergy or reflux and respond to some extent to specific therapies for these conditions. Those who do not respond to empirical therapy should receive workup for the superior laryngeal nerve or the recurrent laryngeal nerve [50]. Lee et al. believe that, as in the case of neuropathic pain [51], there may be a role for neuroleptic medication in the treatment of neuromuscular disorders of the larynx. Open label studies of drugs acting on peripheral nerves used to treat neuropathic pain, such as amitriptyline and gabapentin, have been reported to reduce cough severity but this issue needs further investigation in controlled trials [52,53]. At present, the level at which amitriptyline interferes in the cough reflex is unknown. Recent pain literature suggests that amitriptyline has a rapid, long-acting nerve-blocking effect which is superior to that of a classic local anesthetic. We hypothesize that amitriptyline lowers the sensory threshold of the afferent nerve endings, thus inhibiting the cough reflex. Since clinical benefit has been described in six patients with exercise-induced vocal cord dysfunction who inhaled the anticholinergic drug ipratropium prior to exercise, we cannot rule out this effect since it is known that amitriptyline is anticholinergic [54].

In conclusion, clinical judgement and symptom ratings support the hypothesis that the laryngeal area is the “new frontier” in CC, as the source of a large number of triggers and paresthesias in patients. Several treatments are available not only for laryngeal neuropathy but also for chronic stimuli such as reflux. Speech pathology treatment is an effective behavioral intervention for chronic cough which persists despite medical intervention, since it improves sensory function. In addition, bearing in mind the frequently coincidental onset of neuropathic cough and LPR in patients with CC, we suggest a double therapy targeting the larynx and GER. Further research will shed more light on this issue, especially when the reflux is non-acidic and/or gaseous. All these treatments required a well-coordinated, multidisciplinary approach involving pulmonologists, otolaryngologists and, gastroenterologists in order to resolve the problem of refractory CC.

## Competing interests

The authors declare that they have no competing interest.

## Authors’ contributions

AGP and IC both contributed to writing the manuscript. Both authors read and approved the final manuscript.

## References

[B1] Irwin RS Bauman MH Bolser LP Boulet LP Braman SS Brightling CE American College of Chest Physicians (ACCP). Diagnosis and management of cough executive summary: ACCP evidence-based clinical practice guidelines Chest 2006 129 Suppl 1 1S 23S 16428686 10.1378/chest.129.1_suppl.1S PMC3345522

[B2] Chung KF Chronic “cough hypersensitivity syndrome”: a more precise label for chronic cough Pulm Pharmacol Ther 2011 24 267 271 10.1016/j.pupt.2011.01.012 21292019

[B3] Murry T Bransky RC Yu K Cukier-Blaj S Duflo S Aviv JE Laryngeal sensory deficits in patients with chronic cough and paradoxical vocal fold movement disorder Laryngoscope 2010 120 1576 1581 10.1002/lary.20985 20564660

[B4] Rees CJ Henderson AH Belafsky PC Postviral vagal neuropathy Ann Otol Rhinol Laryngol 2009 118 247 252 19462843 10.1177/000348940911800402

[B5] Pacheco A Faro V Cobeta I Molyneux I Morice AH Gastro-oesophageal reflux, eosinophilic airway inflammation and chronic cough Respirology 2011 16 994 999 10.1111/j.1440-1843.2011.02010.x 21651646

[B6] Haque RA Usmani OS Barnes PJ Chronic idiopathic cough: a discrete clinical entity? Chest 2005 127 1710 1713 10.1378/chest.127.5.1710 15888850

[B7] Everett CF Morice AH Clinical history in gastroesophageal cough Respir Med 2007 101 345 348 10.1016/j.rmed.2006.05.006 16787744

[B8] Irwing R Diagnosis and management of cough: ACCP evidence-based clinical practice guidelines Chest 2006 129 245 254 10.1378/chest.129.1_suppl.25S 16428688

[B9] Morice AH Fontana GA Sovijari AR Pistolesi M Chung KT Widdicombe J ERS Task Force. The diagnosis and management of chronic cough Eur Respir J 2004 24 481 492 10.1183/09031936.04.00027804 15358710

[B10] Vertigan AE Theodoros DG Gibson PG Winkworth AL Efficacy of speech pathology management for chronic cough: a randomised placebo controlled trial of treatment efficacy Thorax 2006 61 1065 1069 10.1136/thx.2006.064337 16844725 PMC2117063

[B11] Vertigan AE Gibson PG Chronic refractory cough as a sensory neuropathy: evidence from a reinterpretation of cough triggers J Voice 2011 25 596 601 10.1016/j.jvoice.2010.07.009 21051202

[B12] Altman KW Simpson CB Amin MR Abaza M Balkissoon R Casiano RR Cough and paradoxical vocal fold motion Otolryngol Head Neck Surg 2002 127 501 511 10.1067/mhn.2002.127589 12501100

[B13] Phua SY McGarvey LP Ngu MC Ing AJ Patients with gastroesophageal reflux disease and cough have impaired laryngopharyngeal mechanosensitivity Thorax 2005 60 488 491 10.1136/thx.2004.033894 15923249 PMC1747421

[B14] Jordan D Central nervous mechanism in cough Pulm Pharmacol 1996 9 389 392 10.1006/pulp.1996.0055 9232683

[B15] Canning BJ Functional implications of the multiple afferent pathways regulating cough Pulm Pharmacol Ther 2011 24 295 299 10.1016/j.pupt.2011.01.008 21272660

[B16] Undem BJ Carr MJ Targeting primary afferent nerves for novel antitussive therapy Chest 2010 137 177 184 10.1378/chest.09-1960 20051402

[B17] Canning BJ Afferent nerves regulating the cough reflex: mechanisms and mediators of cough in disease Otolaryngol Clin N Am 2010 43 14 25 10.1016/j.otc.2009.11.012 PMC2882535 20172253

[B18] Canning BJ Mori N Mazzone SB Vagal afferent nerves regulating the cough reflex Respir Physiol Neurobiol 2006 152 3 223 242 10.1016/j.resp.2006.03.001 16740418

[B19] Mazzone SB Mori N Canning BJ Synergistic interactions between airway afferent nerve subtypes regulating the cough reflex in guinea-pigs J Physiol 2005 569 559 573 10.1113/jphysiol.2005.093153 16051625 PMC1464254

[B20] Morice AH Review article: reflux and airway disease Aliment Pharmacol Ther 2011 33 1 48 52

[B21] Fouad YM Katz PO Hatlebakk JG Castell DO Ineffective esophageal motility: the most common motility abnormality in patients with GERD-associated respiratory symptoms Am J Gastroenterol 1999 94 1464 1467 10.1111/j.1572-0241.1999.1127_e.x 10364008

[B22] Kawamura O Aslam M Rittmann T Hofmann C Shaker R Physical and pH properties of gastroesophagopharyngeal refluxate: a 24-hour simultaneous ambulatory impedance and pH monitoring study Am J Gastroenterol 2004 99 1000 1010 10.1111/j.1572-0241.2004.30349.x 15180717

[B23] Pacheco-Galván A Hart SP Morice AH Relationship between gastro-oesophageal reflux and airway diseases: The airway reflux paradigm Arch Bronconeumol 2011 47 195 203 21459504 10.1016/j.arbres.2011.02.001

[B24] Knight RE Wells JR Parrish RS Esophageal dysmotility is an important co-factor in extraesophageal manifestations of gastroesophageal reflux Laryngoscope 2000 110 1462 1466 10.1097/00005537-200009000-00010 10983943

[B25] Kastelik JA Redington AE Aziz I Buckton GK Smith CM Dakkak M Abnormal oesophageal motility in patients with chronic cough Thorax 2003 58 699 702 10.1136/thorax.58.8.699 12885989 PMC1746758

[B26] Kahrilas PJ Shaheen NJ Vaezi MF Hiltz SW Black E Modlin IM American gastroenterology association medical position statement on the management of gastroesophageal reflux disease Gastroenterolgy 2008 135 1383 1391 10.1053/j.gastro.2008.08.045 18789939

[B27] Vertigan AE Gibson PG Theodoros DG Winkworth AL A review of voice and upper airway function in chronic cough and paradoxical vocal cord movement Curr Opin Allergy Clin Immunol 2007 7 37 42 10.1097/ACI.0b013e328012c587 17218809

[B28] Greene SM Simpson CB Evidence for sensory neuropathy and pharmacologic management Otolaryngol Clin North Am 2010 42 67 72 20172257 10.1016/j.otc.2009.11.003

[B29] Morrison M Rammage L Emami AJ The irritable larynx syndrome J Voice 1999 13 447 455 10.1016/S0892-1997(99)80049-6 10498060

[B30] Pacheco A Wagner C Nieto R García L Jurkojc C Gotera C Multiple dimension of chronic cough. An analysis of 270 cases Arch Bronconeumol 2012 48 Espec Cong 1 29623116900

[B31] McGarvey L McKeagney P Polley L MacMahon J Costello RW Are there clinical features of a sensitized cough reflex? Pulm Pharmacol Ther 2009 22 59 64 10.1016/j.pupt.2008.11.003 19049891

[B32] Amin MR Koufman JA Vagal neuropathy after upper respiratory infections: a viral etiology Am J Otolryngol 2001 22 251 256 10.1053/ajot.2001.24823 11464321

[B33] Kok C Kennerson ML Spring PJ Ing AJ Pollard JD Nicholson GA A locus for hereditary sensory neuropath with cough and gastroesophageal reflux on chromosome 3p22-p24 Am J Human Genet 2003 73 632 637 10.1086/377591 12870133 PMC1180687

[B34] Spring PJ Kok C Nicholson GA Ing AJ Spies JM Bassett ML Autosomal dominant hereditary sensory neuropathy with chronic cough and gastro-esophageal reflux: clinical features in two families linked to chromosome 3p22-p24 Brain 2005 128 2797 2810 10.1093/brain/awh653 16311270

[B35] Taramarcaz P Grissell TV Borgas T Gibson P Transient postviral vocal cord dysfunction J Allergy Clin Immunol 2004 114 1431 1432 10.1016/j.jaci.2004.08.038 15577854

[B36] Blager F Paradoxical vocal fold movement: diagnosis and management Curr Opin Otolaryngol Head Neck Sur 2000 8 180 183 10.1097/00020840-200006000-00009

[B37] Cukier-Blaj S Bewley A Aviv JE Murry T Paradoxical vocal fold motion:a sensory motorlaryngeal disorder Laryngoscope 2008 118 2 367 370 10.1097/MLG.0b013e31815988b0 18000464

[B38] Belafsky PC Postma GN Koufman JA The validity and reliability of the reflux finding score Laryngoscope 2001 111 1315 1317 10.1097/00005537-200108000-00001 11568561

[B39] Patterson N Mainie I Rafferty G McGarvey L Heaney L Tutuian R Nonacid reflux episodes reaching the pharynx are important factors associated with cough J Clin Gastroenterol 2009 43 414 419 10.1097/MCG.0b013e31818859a3 19197196

[B40] Johnston N Knight J Dettmar PW Lively MO Koufman J Pepsin and carbonic anhydrasa isoenzyme III as diagnostic markers for laryngopharyngeal reflux disease Laryngoscope 2004 114 2129 2134 10.1097/01.mlg.0000149445.07146.03 15564833

[B41] Johnston N Dettmar PW Bishwokarma B Livel MO Koufman JA Activity /stability of human pepsin: implications for reflux attributed laryngeal disease Laryngoscope 2007 117 1036 1039 10.1097/MLG.0b013e31804154c3 17417109

[B42] Zang G Lehman A Rigda R Dent J Holloway RH Control of transient lower oesophageal sphincter relaxations and reflux by the GABA (B) in patients with baclofen agonist gastro-oesophageal reflux disease Gut 2002 50 19 24 10.1136/gut.50.1.19 11772961 PMC1773078

[B43] Altman KW MD Haines GK III Hammer ND 3rd Radosevich JA The H+/K+-ATPase (proton) pump is expressed in human laryngeal submucosal glands Laryngoscope 2003 113 1927 1930 14603049 10.1097/00005537-200311000-00013

[B44] Canning BJ Chou YL Cough sensors. Physiological and pharmacological properties of the afferent nerves regulating cough Handb Exp Pharmacol 2009 187 23 47 10.1007/978-3-540-79842-2_2 18825334 PMC13471087

[B45] Groneberg DA Niimi A Dinh QT Cosio B Hew M Fischer A Chung KF Increased expression of transient receptor potential vanilloid-1 in airway nerves of chronic cough Am J Respir Crit Care Med 2004 170 1276 1280 10.1164/rccm.200402-174OC 15447941

[B46] Zhang G Lin RL Wiggers M Snow DM Lee LY Altered expression of TRPV1 and sensitivity to capsaicin in pulmonary myelinated afferents following chronic airway inflammation in the rat J Physiol 2008 586 Pt23 5771 5786 18832423 10.1113/jphysiol.2008.161042 PMC2655410

[B47] Prudon B Birring SS Vara DD Hall AP Thompson JP Pavord ID Cough and glottic-stop reflex sensitivity in health and disease Chest 2005 127 550 557 10.1378/chest.127.2.550 15705995

[B48] Morice AH McGarvey LP Dicpinigaitis PV Cough hypersensitivity syndrome is an important clinical concept: a pro/con debate Lung 2012 190 3 9 10.1007/s00408-011-9351-y 22186805

[B49] Birring SS Controversies in the evaluation and management of chronic cough Am J Respir Crit Care Med 2011 183 708 715 10.1164/rccm.201007-1017CI 21148722

[B50] Lee B Woo P Chronic cough as a sign of laryngeal sensory neuropathy: diagnosis and treatment Ann Otol Rhinol Laryngol 2005 114 253 257 15895778 10.1177/000348940511400401

[B51] Cheshire WP Jr Defining the role for gabapentine in the treatment of trigeminal neuralgia: a retrospective study J Pain 2002 3 137 142 10.1054/jpai.2002.122944 14622800

[B52] Jeyakumar A Brickman TM Haben M Effectiveness of amitriptyline versus cough suppressants in the treatment of chronic cough resulting from postviral vagal neuropathy Laryngoscope 2006 116 2108 2112 10.1097/01.mlg.0000244377.60334.e3 17146380

[B53] Mintz S Lee JK Gabapentin in the treatment of intractable idiopathic chronic cough: case reports Am J Med 2006 119 e13 e15 16651037 10.1016/j.amjmed.2005.10.046

[B54] Weinberger M Abu-Hasan M Pseudo-asthma: when cough, wheezing and dyspnea are not asthma Pediatrics 2007 120 855 864 10.1542/peds.2007-0078 17908773

